# Clinical efficacy of sublingual immunotherapy is associated with restoration of steady-state serum lipocalin 2 after SLIT: a pilot study

**DOI:** 10.1186/s40413-018-0201-8

**Published:** 2018-10-01

**Authors:** Franziska Roth-Walter, René Schmutz, Nadine Mothes-Luksch, Patrick Lemell, Petra Zieglmayer, René Zieglmayer, Erika Jensen-Jarolim

**Affiliations:** 1 Comparative Medicine, The Interuniversity Messerli Research Institute of the University of Veterinary Medicine Vienna, Medical University Vienna and University Vienna, Vienna, Austria; 2 Biomedical International R+D GmbH, Vienna, Austria; 3 Vienna Challenge Chamber, Vienna, Austria; 4 AllergyCare, Allergy Diagnosis and Study Center, Vienna, Austria; 5 Institute of Pathophysiology and Allergy Research, Center of Pathophysiology, Infectiology and Immunology, Medical University of Vienna, Vienna, Austria

**Keywords:** Lipocalin 2, Sublingual immunotherapy, Allergen, Clinical efficacy, Innate

## Abstract

**Background:**

So far, only a few biomarkers in allergen immunotherapy exist that are associated with a clinical benefit. We thus investigated in a pilot study whether innate molecules such as the molecule lipocalin-2 (LCN2), with implications in immune tolerance demonstrated in other fields, may discriminate A) between allergic and non-allergic individuals, and B) between patients clinically responding or non-responding to sublingual allergen immunotherapy (SLIT) with house dust mite (HDM) extract. Moreover, we assessed haematological changes potentially correlating with allergic symptoms.

**Methods:**

LCN2-concentrations were assessed in sera of healthy and allergic subjects (*n* = 126) as well as of house dust mite (HDM) allergics before and during HDM- sublingual immunotherapy (SLIT) in a randomized, double-blind, placebo-controlled trial for 24 weeks. Sera pre-SLIT (week 0), post-SLIT (week 24) and 9 months after SLIT were assessed for LCN2 levels and correlated with total nasal symptom scores (TNSS) obtained during chamber challenge at week 24 in patients receiving HDM- (*n* = 31) or placebo-SLIT (*n* = 10).

**Results:**

Allergic individuals had significantly (*p* < 0.0001) lower LCN2-levels than healthy controls. HDM-allergic patients who received HDM-SLIT showed a significant increase in LCN2 9 months after termination of HDM-SLIT (*p* < 0.001), whereas in subjects receiving placebo no increase in LCN2 was observed. Among blood parameters a lower absolute rise in the lymphocyte population (*p* < 0.05) negatively correlated with symptom improvement (Pearson r 0.3395), and a lower relative increase in the neutrophils were associated with improvement in TNSS (*p* < 0.05). LCN2 levels 9 months after immunotherapy showed a low positive correlation with the relative improvement of symptoms (Pearson r 0.3293). LCN2-levels 9 months off-SLIT were significantly higher in patients whose symptoms improved during chamber challenge than in those whose symptoms aggravated (*p* < 0.01).

**Conclusion:**

Serum LCN2 concentrations 9 months off-SLIT correlated with clinical reactivity in allergic patients. An increase in the LCN2 levels 9 months after HDM-SLIT was associated with a clinical benefit. Serum LCN2 may thus contribute to assess clinical reactivity in allergic patients.

**Trial registration:**

Part of the data were generated from clinicaltrials.gov Identifier NCT01644617.

## Background

The prevalence of allergy is rising in the westernized world affecting already about 35% of all women and 24% of the men in Germany [1]. Similarly, in the United States the prevalence for respiratory allergies has increased to 20%, for food allergies to 5% and for skin allergies to 12% [2]. The reason for the rise in allergies is unclear.

Much focus is given on the deviation of the adaptive immune response in allergic and atopic patients, characterized by a dominant Th2 response and IgE antibodies to harmless allergens. Allergen-specific immunotherapy (AIT) is the only causative treatment against type I allergies and results in profound immunological changes.

AIT in daily practice - for pollen, pet dander, house dust mite, and venom allergies - is mainly applied subcutaneously or sublingually and is suitable for both children and adults [3]. Intralymphatic, percutaneous or oral routes are still under clinical evaluation [4]. Main clinical outcome is a decrease in disease severity, less drug usage and a long-term curative effect. Usually, during allergen immunotherapy an early transient increase with a gradual late decrease or no change in allergen-specific IgE is observed, which is accompanied with an early and continuous increase in specific IgG, especially IgG4. Moreover, allergen-specific Treg and Breg cells are generated and reduced mast cell and basophil activity is observed. A general decrease in mast cell and eosinophil numbers and release of their mediators results then months later in a decrease in type I skin reactivity [5]. However, inhibition of late phase skin reactions already seems to manifest as soon as 2 to 4 weeks after starting immunotherapy, thereby preceding inhibition of early responses by months [6–8]. Importantly this suppression of the late response also precedes the appearance of serologic inhibitory antibody activity and seem to be accompanied by an early induction of IL10 [6].

Although AIT is largely effective, the degree of remission strongly varies depending on the intricate associations of individual patient, type of specific allergen, symptoms and on the type of vaccine used in AIT. To date, there is no consensus on candidate surrogate biomarkers of efficacy that would be prognostic, predictive and/or surrogate of the clinical response to AIT. As such, allergen-specific IgG4 is rather a biomarker for compliance than of effective treatment [9]. Functional assays such as FAB inhibition assessing humoral IgE inhibitory factors seem to better predict clinical efficacy of immunotherapy treatment [10].

Beside an inherited risk [11] and some molecular features of the allergens per se [12–14], especially a lower exposure to microbes [15], seem to be decisive for the rise in allergies. Lower exposure to microbial products [16] and an imbalanced microbiota [17–19] also seem also to promote the innate immune deviation in allergies. In this respect, it seems of interest that in fact allergics have a deviated innate immune response, with a decreased expression of natural and antimicrobial molecules like S100A7 [20], PLUNC proteins [21], calprotectin [21, 22], CC10 [23] and trefoil factor family TFF − 1 [24]. Importantly, upregulation of some innate proteins like lipocalin 2, LCN2, has been implicated to have a protective function at least in mice [25]. LCN2 is usually secreted at mucosal surfaces, but also neutrophils and antigen presenting cells like macrophages and dendritic cells have been implied to express LCN2 [26, 27]. LCN2 contributes to innate immunity and limits bacterial growth by binding to iron-containing siderophores. It can regulate immune cells by acting in a pro- or anti- apoptotic manner dependent on its load [11] and consequently has been proposed to contribute in allergic sensitization [14].

We aimed to assess LCN2 levels in healthy individuals and in allergic patients but also in subjects undergoing AIT. Our hypothesis was that allergics, being deficient in their innate immune response, also must have lower LCN2 in serum than non-allergic controls, likely associated with aberration of other haematological and serum parameters. We investigated patients allergic to house dust mite (HDM) from a single-site double-blind, placebo-controlled, randomized trial of sublingual immunotherapy (SLIT) to house-dust mite extract or placebo. From each subject, allergic reactivity to HDM was assessed in an environmental exposure chamber and their symptoms were objectified by assessing total nasal symptom scores (TNSS) before and after treatment. As such, we were in the position to correlate haematological changes with a clinical benefit.

## Methods

### Sample cohorts

The first cohort included samples of 126 subjects, which were sub grouped into allergic (*n* = 63) or non-allergic subjects (*n* = 46) according to allergen-specific IgE, positive skin prick tests and a positive clinical history of allergic rhinitis. Subjects with asthma and atopic dermatitis were excluded (*n* = 17). Subjects with unspecified symptoms and without specific IgE as well as negative skin prick test were allocated to the non-allergic control group (*n* = 46).

The second study cohort included allergic rhinitis patients from a randomized, placebo-controlled, double-blind trial (NCT01644617). Thirty-one (31) allergics underwent SLIT with tablets of house dust mite (HDM) extract (SLIT), and 10 received placebo for 24 weeks. All subjects underwent environmental exposure chamber challenges with HDM in the Vienna Challenge Chamber [28] at baseline and at week 24. Subjects’ demographic, blood parameter, TNSS and IgE to house dust mite were collected before and after treatment [29]. Additional serum samples were obtained from a subgroup of former study participants, who visited the study site approximately 9 months later. Thus, only subjects, who donated serum 9 month off-SLIT, were included in the present study.

### Analysis of haematological and chemistry parameters

Fifteen routine haematological and sixteen blood chemistry parameters were evaluated from subjects of the HDM-SLIT trial. Median values of each laboratory parameters after termination of SLIT (V9, visit 9 at week 24) or changes of the laboratory parameters before and after SLIT (ΔV9-SCR) were evaluated in subjects treated with house dust mite SLIT tablets or Placebo. Additionally, all study subjects were grouped according to their clinical outcome by setting the threshold to 20% for amelioration of symptoms calculated as (ΔTNSS_after-before_/TNSS_before_*100), irrespective of whether the subjects belonged to the placebo- or active- treated group.

### Determination of LCN2

LCN2-levels were detected with commercially available kit against human LCN2 (R&D Systems, Minneapolis, MN, USA) according to the manufacturers’ protocol using 1:200 diluted sera. Sensitivity of LCN2 assay is reported to be about 75 pg/ml.

### Statistical analysis

Parameters were analyzed with two-tailed Student’s t-test. To analyse differences of LCN2-concentrations at different time points, one-way ANOVA with Tukey’s multiple comparisons test for post-hoc analyses were employed. Data analysis was done with GraphPad Prism 7.0c software (GraphPad, San Diego, CA, USA). Correlation coefficients were obtained using Pearson’s rank method. Two-sided *P*-values are presented and a *p*-value ≤ 0.05 was considered statistically significant.

## Results

### Allergics and non-allergics

#### Allergics have lower serum LCN2-levels than non-allergic controls

**Fig. 1:**
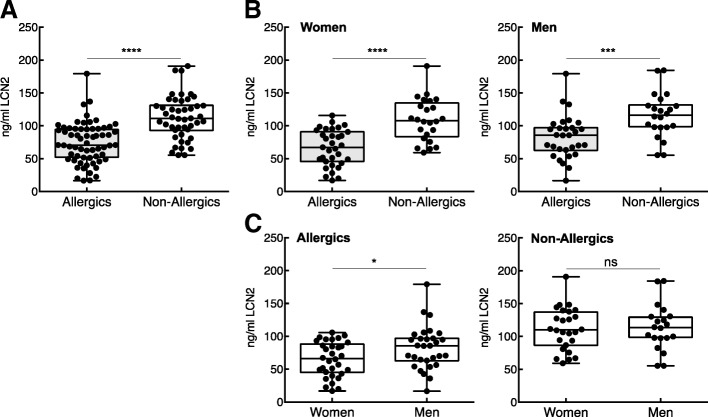
Decreased serum LCN2 levels in allergic compared to non-allergic subjects. Serum LCN2 concentrations were assessed in (**a**) allergic (*n* = 63) and non-allergic (*n* = 46) individuals and (**b**) assessed by gender. **c** Within the allergic cohort, allergic women had lower LCN2-levels than allergic men, whereas no gender-disparity was observed in the non-allergic cohort. Statistical analyses were conducted with Student’s t-test. *p < 0.05, ***p < 0.001, ****p < 0.0001

Allergics with a history of allergic rhinoconjunctivitis had significantly lower LCN2-concentrations in their blood compared to non-allergics, and this was also true when data were analysed by gender (Fig. 1a and b). In our patient cohort, female allergics had significantly lower LCN2-levels, than male allergics. In contrast, no gender differences were observed in the non-allergic group.

### SLIT and placebo

#### Sera and blood parameter changes of active or placebo-treated subjects

We next analysed serum samples and blood parameters of house dust mite allergic subjects from a single-site double blind placebo-controlled SLIT trial in which changes in TNSS were objectified in a challenge chamber [29, 30].

The efficacy and safety outcome of the entire treatment groups are described in detail elsewhere [30]. Subjects characteristics such as age, Der f-specific IgE and symptoms before and after the treatment of obtained samples are depicted in Table 1. By the end of treatment, symptoms significantly ameliorated in the active compared to the placebo-treated groups. A rise in Der f-specific IgE antibodies was observed by the end of the active treatment course at week 24, demonstrating specific immunological reactivity due to house dust mite SLIT tablets, which was not observed in the placebo group.

**Table 1:** Demographics, specific IgE and symptom scores in placebo and active treated group

Parameter Median (Min, Max)	Placebo, *n* = 10	Active, *n* = 31	t test
Age, y	31.4 (20.5–34.6)	24.7 (20.2–53.4)	0.859
women/men	4/6	18/12	
Before SLIT			
Der f - IgE, kUA/l	17.1 (2.8,67.9)	14.3 (0.9, > 100)	0.574
TNSS	6.8 (5.1, 10.3)	7.1 (3.8, 11.8)	0.5213
After SLIT			
Der f - IgE, kUA/l	22.9 (2.2, 45.8)	25.8 (1.3, > 100)	0.096
TNSS	7.7 (4.1,12)	4.1 (0.5, −10.6)	0.0023
Absolute change (after-before)			
Der f - IgE, kUA/l	0.4 (−40.3, 11.8)	9.1 (−14.5, 84.3)	0.007
TNSS	-1 (−2.3, 4.1)	4 (−3.2, 8.5)	0.0021

*TNSS* total nasal symptom score

**Fig. 2:**
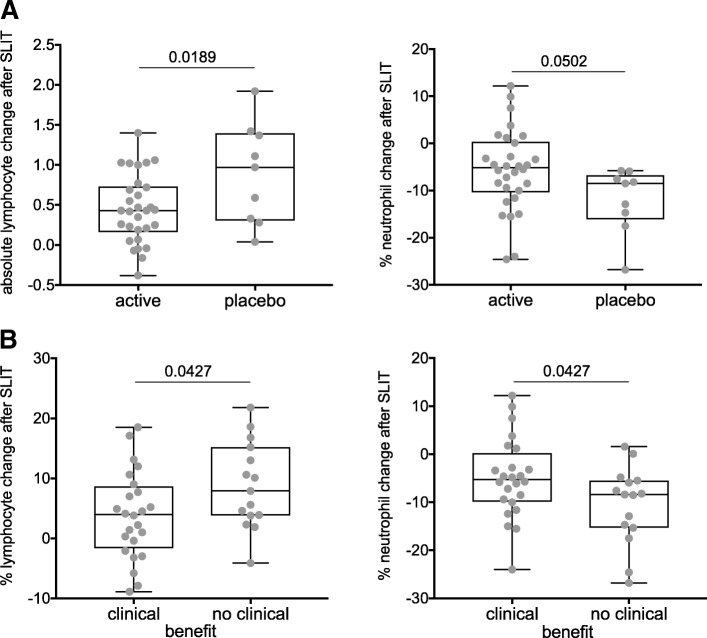
Immunological changes of subjects undergoing sublingual immunotherapy, SLIT. Individual changes in the (**a**) absolute lymphocyte and relative neutrophil counts in active or placebo-treated participants, (**b**) in the relative lymphocyte and neutrophil counts of individuals benefitting or not from sublingual treatment according to TNSS. Statistical analyses were conducted with Student’s t-test

Fifteen haematological and sixteen blood chemistry parameters were assessed. Median values before and after sublingual treatment as well as absolute changes of the parameters are presented in Table 2. While absolute values were similar in the active and placebo treated group after treatments, individual blood parameter changes differed between these two groups. As depicted in Fig. 2a, the active group had a lower absolute increase of lymphocytes and lower relative decrease of neutrophils than the placebo-treated group.

**Table 2:** Blood parameters in placebo and active SLIT groups and their absolute change

Hematology	Before SLIT		T-test	After SLIT		t-test	Absolute change (after-before)		
	Placebo, *n* = 10	Active, *n* = 31		Placebo, *n* = 10	Active, *n* = 31		Placebo, *n* = 10	Active, *n* = 31	
	Median (Min, Max)	Median (Min, Max)		Median (Min, Max)	Median (Min, Max)		Median (Min, Max)	Median (Min, Max)	t-test
red blood cell count [10″ 12/L]	4.9 (4, 5.3)	4.5 (3.9, 5.3)	0.190	5.2 (4.3, 5.6)	4.55 (3.8, 5.5)	0.017	0.3 (−0.4, 0.6)	0.100 (− 0.5, 0.4)	0.062
hematocrit	0.441 (0.363, 0.487) 0.424 (0.349, 0.484)		0.379	0.47 (0.367,0.509)	0.429 (0.363,0.507)	0.131	3.026 (−0.036,0.052)0.013 (− 0.041,0.037)		0.367
hemoglobin [g/L]	148 (113, 161)	141 (111, 171	0.458	149 (113, 169)	139 (114, 167)	0.136	2 (−15, 14)	-1 (−18,8)	0.299
platelete count [10^A^9/L]	259 (164, 329)	229 (168, 381)	0.582	242 (159, 378)	235 (155, 361)	0.532	5 (−107, 104)	2 (−96, 62)	0.635
white cell count [10^A^9 /L]	5.75 (3.7, 15.2)	5.7 (3.1, 12.3)	0.465	6.4 (5.3, 9.3)	6 (4.3, 12.5)	0.763	1.1 (−9.4, 2.7)	0.3 (−4, 4.1)	0.579
basophils abs [10^A^9/L]	0.02 (0.01,0.05)	0.03 (0, 0.08)	0.413	0.03 (0,0.05)	0.03 (0.01,0.07)	0.530	0 (−0.02, 0.03)	0.00 (− 0.02, 0.03)	0.768
basophils [%]	0.3 (0.2, 1.1)	0.5 (0.1, 1.1)	0.359	0.5 (0.1, 0.6)	0.5 (0.2, 1)	0.467	0.1 (−0.6, 0.3)	0.0 (−0.6, 0.7)	0.920
eosinophils abs [10^A^9/L]	0.09 (0, 0.3)	0.14 (0.04, 1.26)	0.215	0.22 (0.07, 0.47)	0.25 (0.12, 0.5)	0.578	0.11 (−0.03,0.37)	0.09 (−0.8, 0.33)	0.357
eosinophils [%]	2 (0.1, 4)	2.5 (0.5,21.5)	0.245	3.5 (1.2, 7)	4.25 (1.7, 9.7)	0.304	1.5 (−2,5.5)	1.25 (−13.6,4.2)	0.615
lymphocytes abs [10^A^9/L]	1.66 (1.19,2.56)	1.73 (1.11,3.03)	0.727	2.77 (1.83, 3.56)	2.15 (1.43, 4.43)	0.114	0.97 (0.04, 1.92)	0.43 (−0.38, 1.4)	0.019
lymphocytes [%]	29.8 (9.9, 41)	30.6 (19.1,47.9)	0.573	38.7 (29.6, 57.8)	37.2 (16.8, 50.7)	0.204	10.1 (−0.4,21.8)	3.85 (−8.9, 18.6)	0.052
monocytes abs [10 ^A^9/L]	0.345 (0.22, 0.62)	0.32 (0.15, 0.89)	0.911	0.39 (0.37, 0.6)	0.38 (0.21,0.89)	0.652	0.04 (−0.07,0.32)	0.03 (−0.47, 0.35)	0.632
monocytes [%]	5.7 (2.9, 10)	5.7 (2.4, 10.4)	0.852	6.4 (4.7, 9.7)	6.55 (3.8, 11.8)	0.744	0.3 (−2.2, 3.6)	0.6 (−5.2, 4.2)	0.745
neutrophils abs [10^A^9/L]	3.55 (1.92, 13.18)	3.33 (1.4, 8.93)	0.310	3.32 (1.7,5.41)	3.31 (1.61,9.14)	0.752	0.22 (−9.71,0.8)	0.04 (−4.87, 3.15)	0.199
neutrophils [%]	62.1 (49.7,86.9)	58.9 (41,72.8)	0.343	53.3 (32.2, 60.1)	50.6 (34.4, 73)	0.330	−8.5 (−26.8,-5.8)	−5.15 (− 24.6, 12.2)	0.050
protein in serum [g/L]	72 (69, 80)	74 (66, 79)	0.846	76 (68, 78)	74 (65, 83)	0.686	1 (−7,6)	1 (− 7, 9)	0.747
albumin [g/L]	48.5 (44, 55)	48 (42, 53)	0.567	52 (43, 55)	48 (39, 53)	0.182	0 (−6, 8)	0 (−5, 6)	0.515
calcium [mmol/L]	2.49 (2.31,2.57)	2.45 (2.3, 2.73)	0.862	2.42 (2.34, 2.64)	2.45 (2.24, 2.58)	0.786	−0.03 (−0.16, 0.14)	−0.01 (−0.31,0.19)	0.812
phosphor inorganic [mmol/L]	1.08 (0.83, 1.57)	1.14 (0.8, 1.3)	0.772	1.24 (1.01, 1.47)	1.21 (0.89, 1.54)	0.502	0.19 (−0.36,0.41)	0.15 (−0.26, 0.43)	0.962
urea nitrogen [mmol/L]	4.5 (3.5, 7.5)	4.4 (2.4, 7.8)	0.631	5.5 (4.4, 6.3)	4.7 (3,7.1)	0.112	0.3 (−1.5, 2.8)	0.2 (−2.4, 3)	0.678
bilirubin [umol/L]	10 (5,20)	10 (4, 35)	0.582	8 (5, 10)	8 (4, 25)	0.123	0 (−12, 2)	−1 (− 10,8)	0.397
alkaline phosphatase [U/L]	55 (43, 69)	62 (31,92)	0.264	60 (47, 86)	65 (29, 104)	0.430	4 (−12, 34)	3 (−8, 38)	0.843
AS AT (SGOT) [U/L]	20 (15, 30)	20 (16, 40)	0.710	20 (12, 36)	19 (13, 35)	0.518	1 (−5, 15)	−1 (−9, 10)	0.240
ALAT (SGPT) [U/L]	12 (7,39)	13 (6, 48)	0.836	14 (7,39)	13 (7,50)	0.635	2 (−4, 24)	−1 (− 14,40)	0.522
lactic dehydrogenase [U/L]	157 (113,200)	148 (111, 178)	0.147	160 (115, 188)	146 (116, 184)	0.472	2 (−34, 22)	5 (−16, 26)	0.224
creatinine [umol/L]	72.6 (65.9, 96.5)	74.3 (56.8, 113.4)	0.599	72 (63.8, 103.1)	71.7 (57.5, 113.3)	0.987	−2.2 (−7.7, 12.1)	−0.1 (− 34, 11.3)	0.500
glucose [mmol/L]	4.9 (3.8, 5.8)	5 (3.7, 6)	0.728	4.8 (3.4, 5.7)	4.9 (3.9, 12.1)	0.317	−0.2 (−1.2, 1)	0.2 (− 1.3, 6.1)	0.480
potassium [mmol/L]	4.45 (4.1, 5.2)	4.4 (3.8, 5.1)	0.158	4.3 (3.9, 5.4)	4.3 (3.9, 5.1)	0.491	−0.2 (−0.9, 1)	− 0.1 (− 0.5, 0.5)	0.392
sodium [mmol/L]	140 (138, 142)	140 (137, 144)	0.111	140 (137, 145)	139 (137, 142)	0.069	−1 (−3, 4)	0 (−4, 3)	0.814
chloride [mmol/L]	104 (101, 106)	103 (100, 106)	0.275	104 (98, 109)	104 (98, 108)	0.733	0 (−4, 5)	1 (−5, 4)	0.952
carbon dioxide, C02 [mmol/L]	21 (17, 24)	21 (17, 25)	0.496	23 (18, 26)	22 (18, 25)	0.077	2 (−3,7)	0 (−6, 6)	0.158

### Responders and non-responders

#### Blood parameter changes according to the clinical benefit of subjects

Obtained samples were thereafter grouped in patients benefitting or not from the treatment irrespective whether the prior belonged to the active or placebo treated group. Here a different picture emerged as 1 subject of the placebo-treated group benefitted and 6 of the active group treated with house dust mite SLIT tablets did not benefit from the treatments. Overall, subjects with more severe symptoms at start of treatment seemed to have benefitted to a greater extent from the sublingual treatment compared to subject with milder symptoms (Table 3).

**Table 3:** Demographics, specific IgE and symptom scores in subjects according to clinical benefit

Parameter Median (Min,Max)	non-responders, *n* = 15	responders, *n* = 26	t test
Age, y	24.4 (20.5, 42.8)	26.4 (20.0, 53.4)	0.859
women/men	9/6	14/12	
Before SLIT			
Der f - IgE, kUA/l	15 (2.8, 67.9)	13 (0.9, > 100)	0.904
TNSS	6.5 (3.8, 10.3)	7.7 (5.4, 11.8)	0.023
After SLIT			
Der f - IgE, kUA/l	24.9 (2.2, > 100)	25.8 (1.3, > 100)	0.303
TNSS	8.1 (4.7, 12.0)	3.8 (0.5, 7.3)	3 × 10^^^-9
Absolute change (after-before)			
Der f - IgE, kUA/l	1.7 (−40.3, 84.3)	6.2 (−14.5, 81.5)	0.310
TNSS	1.5 (−1.3,3.2)	−4.2 (−8.5, − 1.8)	2 × 10^^^-14
% improvement	−21 (−56, 18)	58 (20, 92)	4 × 10^^^-15

TNSS total nasal symptom score

**Fig. 3:**
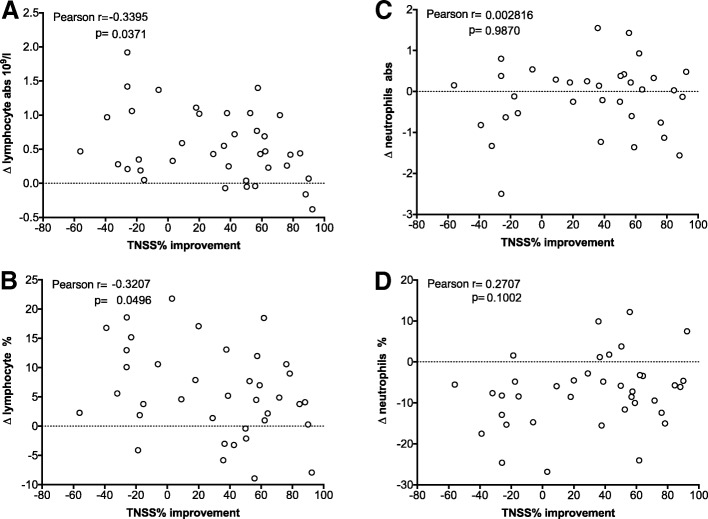
Correlation of relative total nasal symptom score (TNSS) improvement with changes in (**a**) absolute lymphocyte, (**b**) relative lymphocyte numbers as well as changes of (**c**) absolute and (**d**) relative neutrophil counts before and after 6 months treatment. Correlation were obtained using Pearson’s rank method

Moreover, individual changes in the absolute lymphocyte and relative neutrophil population became apparent. Also, here a clinical benefit was associated with a lower relative rise of the lymphocyte population and a lower relative decrease of blood neutrophils in responders compared to non-responders (Fig. 2b and Table 4). As depicted in Fig. 3, when changes in the relative and absolute lymphocyte were correlated with clinical improvement, a significant small negative correlation with the lymphocytes - relative and absolute – became apparent. Absolute changes in neutrophil number did not correlate at all, though a positive, not significant, trend in relative neutrophil changes with symptom improvement were observed.

**Table 4:** Blood parameters of subjects according to their clinical benefit after treatment

Hematology	Before SLIT		After SLIT			Absolute change (after-before)			
	no benefit, *n* = 15	clinical benefit, *n* = 26		no benefit, *n* = 15	clinical benefit, *n* = 26		no benefit, *n* = 15	clinical benefit, *n* = 26	
	Median (Min, Max)	Median (Min, Max)	t-test	Median (Min, Max)	Median (Min, Max)	t-test	Median (Min, Max)	Median (Min, Max)	t-test
red blood cell count [10^A^12/L]	4.6 (4, 5.3)	4.6 (3.9, 5.3)	0.773	4.8 (3.8, 5,6)	4.7 (3.8, 5.5)	0.663	0.2 (−0.4, 0.6)	0.1 (−0.5, 0.4)	0.297
hematocrit	0.432 (0.349, 0.487)	0.429 (0.376, 0.482)	0.699	0.437 (0.367, 0.509)	0.433 (0.363, 0.507)	0.886	0.016 (−0.036, 0.052	0.013 (−0.041, 0.034)	0.586
hemoglobin [g/L]	144 (111, 171)	142 (125, 162)	0.735	145 (113, 169)	139 (114, 167)	0.943	0 (−15, 14)	0.5 (−18, 7)	0.716
platelete count [10^A^9/L]	268 (164, 381)	229 (168, 373)	0.225	243 (155, 378)	231 (177, 361)	0.460	−5 (−107, 104)	2.5 (−71, 36)	0.670
white cell count [10^A^9 /L]	5.55 (3.7, 15.2)	5.7 (3.1, 12.3)	0.706	6.1 (4.3, 12.5)	6.1 (4.3, 10.9)	0.795	0.9 (−9.4, 4.1)	0.25 (−4, 3.8)	0.853
basophils abs [10A9/L]	0.025(0.01, 0.05)	0.02 (0, 0.08)	0.975	0.03 (0.01, 0.05)	0.03 (0, 0.07)	0.524	0 (−0.02, 0.03)	0 (−0.02, 0.02)	0.442
basophils [%]	0.35 (0.2, 1,1)	0.5 (0.1, 1.1)	0.731	0.5 (0.2, 1)	0.5 (0.1, 0.8)	0.453	0 (−0.6, 0.7)	0 (−0.6, 0,5)	0.438
eosinophils abs [10A9/L]	0.15 (0, 0.42)	0.13 (0.04, 1.26)	0.559	0.25 (0.07, 0.5)	0.245 (0.12, 0.47)	0.965	0.08 (−0.04, 0.27)	0.09 (−0.8, 0.37)	0.569
eosinophils [%]	2.65 (0.1, 6.2)	2.3 (0.5, 21.5)	0.604	4.2 (1.2, 9.7)	4.2 (1.7, 7.9)	0.846	1.5 (−2, 4.2)	1.25 (−13.6, 5.5)	0.551
lymphocytes abs [10A9/L]	1.67 (1.19, 2.56)	1.76 (1.11, 3.03)	0.407	2.43 (1.71, 3.56)	2.17 (1.43, 4.43)	0.463	0.59 (0.05, 1.92)	0.43 (−0.38, 1.4)	0.082
lymphocytes [%]	29.9 (9.9, 41)	30.6 (19.1, 47.9)	0.393	38.7 (16.8, 57.8)	36.9 (23.3, 50.7)	0.323	7.9 (−4.1, 21.8)	3.95 (−8.9, 18.5)	0.043
monocytes abs [10A9/L]	0.375 (0.15, 0.62)	0.31 (0.23, 0.89)	0.936	0.39 (0.3, 0.89)	0.385 (0.21, 0.71)	0.499	0.01 (−0.14, 0.35)	0.04 (− 0.47, 0.32)	0.603
monocytes [%]	6.5 (2.4, 10.4)	5.6 (4.1, 9)	0.742	6.5 (4.7, 9.7)	6.45 (3.8, 11.8)	0.603	0.3 (−4.5, 4.2)	0.8 (−5.2, 3.6)	0.926
neutrophils abs [10A9/L]	3.34 (1.92, 13.18)	3.33 (1.4, 8.9)	0.526	3.18 (1.61, 9.14)	3.57 (1.63, 6.38)	0.929	0.15 (−9.71, 3.15)	0.04 (−4.87, 2.72)	0.476
neutrophils [%]	60.4 (49.7, 86.9)	58.9 (41, 72.8)	0.392	50.6 (32.2, 73)	51.4 (35.9, 68,3)	0.301	−8.4 (−26.8, 1.6)	−5.25 (−24, 12.2)	0.043
protein in serum [g/L]	73 (66, 80)	73 (66, 79)	0.869	76 (67, 78)	74 (65, 83)	0.866	1 (−7, 6)	1 (−5, 9)	0.767
albumin [g/L]	48 (43, 55)	48 (42, 53)	0.830	47 (43, 55)	48 (39, 53)	0.768	0 (−6, 8)	0 (−5, 6)	0.914
calcium [mmol/L]	2.46 (2.31, 2.73)	2.47 (2.3, 2.57)	0.477	2.42 (2.28, 2.64)	2.46 (2.24, 2.58)	0.874	−0.01 (−0.31, 0.14)	−0.01 (−0,16, 0.19)	0.314
phosphor inorganic [mmol/L]	1.08 (0.8, 1.57)	1.17 (0.82, 1.3)	0.677	1.24 (1.01, 1.51)	1.21 (0.89, 1.54)	0.407	0.19 (−0.36, 0.43)	0.14 (−0.26, 0.41)	0.431
urea nitrogen [mmol/L]	4.4 (2.6, 7.8)	4.6 (2.4, 7.3)	0.783	4.7 (3.3, 6.3)	4.9 (3, 7.1)	0.767	0.3 (−1.6, 2.8)	0.2 (−2.4, 3)	0.835
bilirubin [umol/L]	9.5 (5, 16)	10 (4, 35)	0.205	8 (4, 13)	8 (4, 25)	0.064	−2 (−9, 5)	−1 (− 12, 8)	0.898
alkaline phosphatase [U/L]	57 (43, 74)	64 (31, 92)	0.253	59 (47, 94)	70 (29, 104)	0.369	2 (−12, 38)	4 (−8, 33)	0.918
ASAT (SGOT) [U/L]	20.5 (15,40)	19 (16, 29)	0.394	20 (12, 36)	18 (13, 30)	0.055	0 (−6, 15)	−1 (−9, 8)	0.211
ALAT (SGPT) [U/L]	12 (6, 48)	13 (7, 34)	0.584	15 (7, 46)	13 (7, 50)	0.134	1 (−11, 40)	0 (−14, 20)	0.277
lactic dehydrogenase [U/L]	157 (113, 200)	148 (111, 178)	0.141	159 (115, 188)	146 (116, 184)	0.640	0 (−34, 22)	5 (−16, 26)	0.137
creatinine [umol/L]	71.6 (59.8, 113.4)	75.9 (56.8, 101.4)	0.588	70.7 (60.7, 113.3)	72.5 (57.5, 103.1)	0.845	−2.2 (−7.7, 12.1)	1.1 (−34, 11.3)	0.662
glucose [mmol/L]	4.8 (3.7, 6)	5 (3.8, 5.8)	0.261	4.9 (3.7, 12.1)	4.9 (3.4, 7.9)	0.281	0.7 (−0.7, 6.1)	−0.1 (−1.3, 2.9)	0.062
potassium [mmol/L]	4.55 (3.9, 5.2)	4.4 (3.8, 5.1)	0.014	4.4 (3.9, 5.4)	4.2 (3.9, 4.9)	0.045	−0.2 (− 0.9, 1)	−0.1 (− 0.5, 0.5)	0.512
sodium [mmol/L]	139 (137, 144)	140 (137, 141)	0.975	140 (137, 141)	139 (137, 145)	0.699	−1 (−4, 3)	0 (− 1, 4)	0.782
chloride [mmol/L]	103 (100, 106)	104 (101, 106)	0.832	104 (98, 106)	104 (100, 109)	0.768	2 (−5, 4)	1 (− 4, 5)	0.958
carbon dioxide, CO2 [mmol/L]	21 (17, 24)	22 (17, 25)	0.388	23 (18, 26)	22 (18, 25)	0.183	^1^ (−3 ^7^)	0 (−6, 6)	0.183

### SLIT and placebo

#### Serum LCN2-levels in allergic subjects are increased 9 months after active SLIT and correspond to improvement in TNSS

**Fig. 4:**
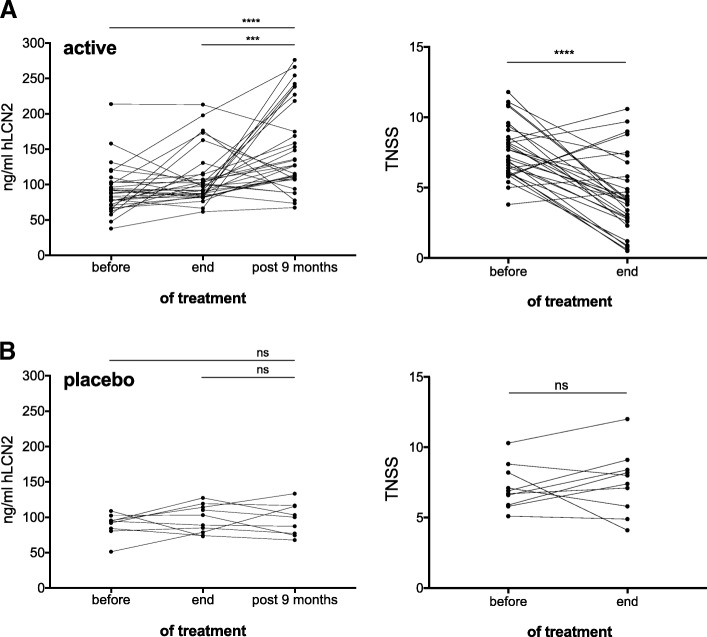
LCN2 serum concentrations rise in the active, but not placebo-treated group approximately 9 months after end of sublingual treatment. LCN2-concentration and total nasal symptom scores (TNSS) in (**a**) active and (**b**) placebo-treated participants. Statistical analyses were conducted using one-way ANOVA using Tukey’s multiple comparisons test for post-hoc analyses. **** *p* < 0.0001, *** *p* < 0.001

**Fig. 5:**
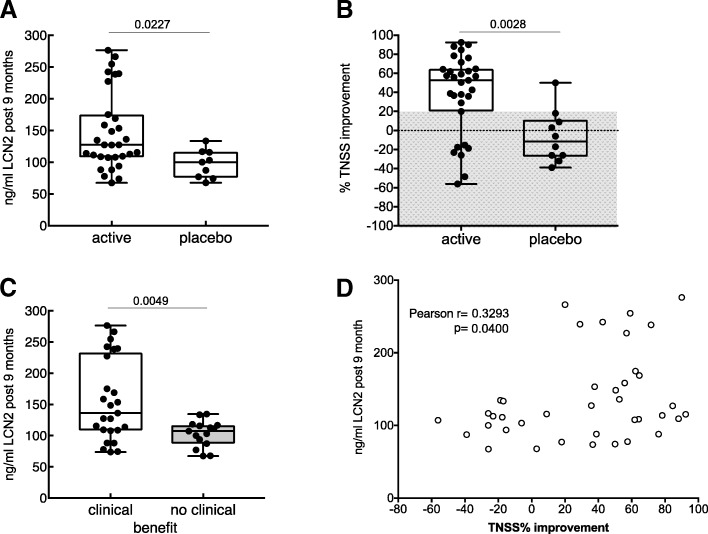
LCN2 concentrations 9 months off-SLIT are higher in subjects whose symptoms ameliorated. (**a**) LCN2-concentration after end of treatment and (**b**) individual relative improvement of the total nasal symptom score, TNSS, in the active and placebo-treated groups. The threshold of 20% for amelioration of symptoms (grey area) were set to group study participants according to their clinical outcome. (**c**) LCN2-concentrations 9 months off-SLIT of patients according to their clinical outcome, irrespective whether the subjects belonged to the placebo- or active treated group. (**d**) Correlation of absolute LCN2-levels 9 month off-SLIT with subjects’ relative improvement in the TNSS. Statistical analyses were conducted using one-way ANOVA using Tukey’s multiple comparisons test for post-hoc analyses. Correlation was obtained using Pearson’s rank method

While LCN2 concentrations did not change significantly during the beginning of the treatment, a highly significant rise of the serum LCN2 levels approximately 8 mos off-SLIT was observed in patients who underwent active sublingual treatment. This phenomenon was not observed in patients of the placebo group (Figs. 4 and 5a).

### Responders and non-responders

**Fig. 6:**
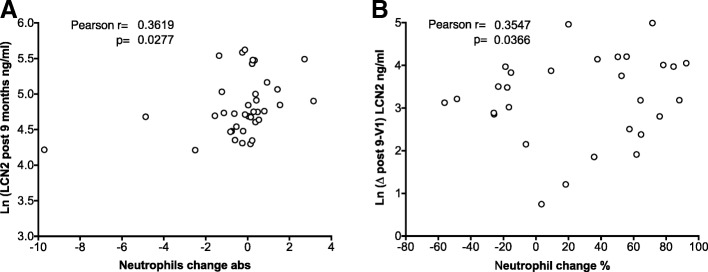
LCN2 correlate with neutrophils. Correlation of natural log-transformed LCN2 concentration occurring 9 months off-SLIT with changes in the (**a**) absolute and (**b**) relative numbers of the neutrophil population. Correlation was obtained using Pearson’s rank method

When study participants were analysed next according to symptoms improvement, it became apparent that LCN2-concentrations 9 months off-SLIT were significantly higher in patients who benefited from SLIT, than in patients whose symptoms did not improve (Fig. 5). Moreover, LCN2 rise 9 months after SLIT correlated significantly with the clinical improvement in patients (Fig. 5d). The source of LCN2 were likely neutrophils as LCN2 changes significantly correlated with absolute and relative changes of the neutrophil population (Fig. 6).

## Discussion

The higher allergy risk has been linked in numerous studies with the lack of pathogen-recognition receptors such as toll like receptors 4 [31, 32], TRIF [33] and MyD88 [16, 33, 34]. Also cytokine-deficiencies such as of interleukin 15, which is produced as a mature protein mainly by dendritic cells, monocytes and macrophages, can exacerbate allergy [35].

Accordingly, and in line with our hypothesis, allergics of our patient cohort had significantly lower serum levels of the innate protein LCN2 than non-allergics.

LCN2 is one of the innate proteins that directly can affect the microbiota as it can sequester bacterial-derived siderophores, which are low molecular compounds with high affinity to iron [36]. Indeed, LCN2 seems to act as a sentinel for bacterial siderophores rather than for iron, with increased siderophore levels resulting in an increase in LCN2 expression. Several studies reported a lower bacterial abundancy and diversity in allergics than non-allergics [17–19], suggesting that a lower number of bacteria secrete lower levels of siderophores and “requiring” lower LCN2-levels in the host to keep the commensal bacteria at bay.

Accordingly, in this study, significantly lower LCN2 levels were measured in allergics. In our patient cohort, levels were lower in allergic women than allergic men, though no gender-difference were was observed in the non-allergic controls. Possible explanations for the gender-bias in allergics may be the link of LCN2 with iron, reflecting a lower iron-status of allergic women compared to the allergic men, causing part of the gender-bias in allergies [37].

In a next step, we followed the course of symptoms in allergics of a double-blind, placebo-controlled trial that underwent treatment with house dust mite SLIT tablets and correlated whether LCN2 or other blood parameters could be correlated with amelioration of symptoms.

Absolute values of blood parameter did not differ neither in the placebo and active treated groups nor in responding or non-responding patients. However, subjects with the active treatment were more resilient to an absolute increase in the lymphocyte-count and a relative decrease in neutrophils than the subjects, who received the placebo tablets.

Allergic individuals whose symptoms ameliorated during treatment with house dust mite SLIT tablets had a smaller absolute increase in the lymphocyte counts, and a smaller relative decrease in neutrophils than allergics not benefitting of the treatment. Importantly, the absolute and relative changes in the lymphocyte numbers correlated moderately with the treatment response: A lower rise in the lymphocyte population correlated with a beneficial response to treatment, whereas in patients not benefitting from the treatment the lymphocyte population expanded to a greater extend. Thus the “resilience” to immune activation clearly suggests an active immune-regulatory mechanism of SLIT.

By the end of SLIT, a relative “resilience” of neutrophils to decrease also was observed in the responder group, suggesting that a relative increase of the neutrophil populations might be beneficial for the allergic patient. This is an interesting finding, as neutrophils are the major source for circulating LCN2 under normal, physiological conditions [38], and which confirms our data showing a modest correlation of LCN2 changes 9 months off-SLIT with changes occurring in the neutrophilic population. In the responder group LCN2-levels did not change during immunotherapy but increased in the following months and correlated with symptom improvement.

We speculate that one of the reasons for the low LCN-levels remaining unchanged upon initiation and during therapy, is the action of the introduced allergens. A great number of allergens exert innate defense functions and are capable of binding to the same ligands as LCN2 [11, 12]. Consequently, during immunotherapy allergens may simulate a lower bacterial burden to LCN2, despite the concurrent changes occurring in the immunological course and microbial repertoire. By the end of treatment, with no further help, neutrophils have to boost their LCN2 production to keep the altered microbiota at bay. Thus, the delayed rise of LCN2 after allergen immunotherapy may indicate a recovery of neutrophilic functions or a change in the commensal microbial compositions in patients with a clinical benefit and may point towards a repair of innate defense mechanism by SLIT.

Taken together, our study did not focus on the classical parameters like antigen-specific IgE [39] and IgG4 [9] antibody levels or cellular markers and changes hereof during AIT, but focused on innate contributing factors that correlate with 1.) an established allergy, and 2.) with improvement of clinical symptoms during allergen immune therapy.

## Conclusions

Our data demonstrate that the innate LCN2 protein is decreased in allergic subjects and that an adjustment to levels present in non-allergic subjects is associated with a clinical benefit. This is in contrast to the up-regulation of LCN2 in diseases such as cancer, which is correlated with an overshooting immune tolerance and where elevated LCN2 levels are used as a clinical biomarker [40, 41].

The determination of lowered steady state serum LCN2 levels in allergic patients and their correction by AIT may thus contribute to assess clinical reactivity in allergics [42].

## Abbreviations

AIT: allergen immunotherapy
HDM: house dust mite extract
LCN2: lipocalin 2
SLIT: sublingual immunotherapy
TNSS: total nasal symptom scores
